# A Comprehensive Scientific Survey of Excipients Used in Currently Marketed, Therapeutic Biological Drug Products

**DOI:** 10.1007/s11095-020-02919-4

**Published:** 2020-09-24

**Authors:** V. Ashutosh Rao, Jennifer J. Kim, Dipti S. Patel, Kimberly Rains, Corey R. Estoll

**Affiliations:** 1 Laboratory of Applied Biochemistry, Division of Biotechnology Review and Research III, Office of Biotechnology Products, Center for Drug Evaluation and Research, Food and Drug Administration, 10903 New Hampshire Ave / Bldg. 52/72 Rm 2212, Silver Spring, Maryland 20993 USA; 2 Present Address: Office of Biotechnology Products, Center for Drug Evaluation and Research, Food and Drug Administration, Silver Spring, MD USA; 3 University of Maryland School of Pharmacy, Baltimore, Maryland USA; 4 Office of Biotechnology Products, Center for Drug Evaluation and Research, Food and Drug Administration, Silver Spring, Maryland USA

**Keywords:** biotechnology, excipients, formulation, lyoprotectant, pH, protein, route of administration, stabilizer, surfactant

## Abstract

***Purpose*:**

The steady development of biotechnology-derived therapeutic biologics over the last few decades has generated drugs that are now standard medical treatments for a range of indications. While the development of protein products has surged in recent years, the formulation and delivery of these complex molecules have relied on drug-specific studies and, in some instances, data from non-proteinaceous drug products. The commonalities, trends, and gaps in excipient technologies used to support the development of therapeutic proteins largely remain unexplored due to the drug-specific nature of many formulations.

***Methods*:**

Using a comprehensive and relational database approach, we aimed to provide a scientific survey of all approved or licensed biotechnology-derived drug products with the goal of providing evidence-based information on common attributes and trending features in protein product excipients. We examined 665 formulations, and 395 unique formulations based on having unique excipients within them, that supported 211 therapeutic proteins as of June 2020.

***Results*:**

We report the prevalence of each excipient class and excipient chemical used in eight different drug types including monoclonal antibodies, antibody conjugates, cytokines and growth factors, enzymes, polypeptide hormones, pulmonary surfactants, recombinant fusion proteins, and toxins. We also report the prevalence by excipient type among all therapeutic proteins, in the context of each drug’s recommended pH range, concentration ranges for excipients, and route of administration.

***Conclusions*:**

The results of our analyses indicate certain excipients common to monoclonal antibodies, cytokines, and polypeptide hormones. We also report on excipients unique to protein drug products, such as amino acids, solubilizers, and lyoprotectants. Overall, our report summarizes the current landscape of excipients used in marketed biotechnology-derived therapeutic biologic products.

## Introduction

Biotechnology-derived drug products are produced from biological sources including human, animal, plant, or microbial and can be composed of proteins, nucleic acids, or a combination (1). Protein molecules are inherently unstable outside of their natural biochemical environment. For this reason, proteins developed as therapeutic agents are carefully characterized and stabilized to deliver the intended quality, safety, and efficacy profile. The stability of therapeutic proteins during storage, handling and use is a topic of intense and rich research efforts by drug product developers (2). Excipients are vital in formulating the dosage form by enhancing the manufacturability, stability, and delivery of the drug product. As complex molecules, proteins present unique considerations as active pharmaceutical ingredients (APIs) due to their large molecular weight, amino acid sequence, higher-order structure, post-translational modifications, co-purifying impurities, binding affinity, and biological activity. Protein formulation development can be especially challenging because, in addition to chemical degradation, protein drugs are susceptible to physical degradation of reversible and irreversible aggregates, often resulting in a significantly altered quality profile and immunological or antigenic reaction in patients (3). Thus, stable protein formulations require that both physical and chemical degradation pathways of the drug product are controlled with the diligent use of excipients (4).

Although increased attention is now paid to excipients used in biotechnology-derived drug products, existing resources and literature on excipients rarely distinguish the use of excipients among different drug types, such as small molecule synthetic drugs and biotechnology-derived protein drugs. Such gaps in literature can drive futile efforts towards stabilizing a protein drug product when data from small molecule drug formulation studies are applied.

We hypothesized that a comprehensive and up-to-date survey of excipients in biological therapeutic products could aid the biopharmaceutical industry by minimizing the time spent on preformulation studies to accelerate the formulation development of promising new therapeutic proteins. The ability to determine commonly used excipients or similarities across protein drug classes can aid in improving the efficiency of the formulation development process by providing evidence-driven data on the excipients associated with a known *versus* unknown quality profile. As well, a comprehensive survey of all excipients used in licensed/approved therapeutic biological products could theoretically provide an opportunity to extrapolate information regarding excipients that may be responsible for similar adverse reactions, interactions, or problematic quality profiles associated with various biotechnology-derived drug products. We also provide this information in the context of other known formulation parameters that impact the stability of the drug product such as concentration, pH, dosage form, and route of administration.

In this report, we present a comprehensive survey of excipients utilized in all currently approved or licensed biotechnology-derived drug products to analyze and identify commonalities and trends among excipients used in the context of the therapeutic protein products they support. The survey includes data on active ingredients, excipients, and dosage form attributes of each drug product, to potentially enable extrapolation of information on multiple pharmaceutically relevant aspects. Our goal is to provide clarity on formulations used in marketed therapeutic proteins and improve evidence-based risk assessment for protein formulations.

## Materials and Methods

### Data Sources

To begin curating a database of excipients used in biotechnology-derived drug products, a list of drug products currently approved or licensed by the Office of Biotechnology Products in the Office of Pharmaceutical Quality, Center for Drug Evaluation and Research (CDER) was generated. CDER’s list of licensed or approved biological products was matched with the Purple Book or the Preliminary List of Approved New Drug Applications (NDAs) for biological products (5,6). The current presentation and composition for each drug product was obtained from current prescribing information, publicly available on the Drugs@FDA website (7). If prescribing information (PI) was unavailable from the website, prescribing information provided by drug manufacturer websites was utilized as a secondary resource. The excipients were analyzed using a relational database program, Microsoft Access (Microsoft Corporation, Office 365 ProPlus, Access version 1902), which allowed analysis of large data through a platform and a user interface for performing targeted queries.

### Data Curation

Since drug products manufactured in multiple drug formulations may differ in quantity or concentration of active ingredients, dosage form, package size or type of excipient, we captured unique drug formulation pertaining to the same API as a unique entry in our collection. To accurately account for these differences, data were organized by drug formulations using the assigned National Drug Code (NDC) rather than by proprietary name or active ingredient name of the drug product (8). Formulations that had the same composition and concentration of active and inactive ingredients with a different package size or type were counted as a single entry for the analysis to minimize duplication of data. For each unique formulation that was identified, all excipient names and respective quantities were recorded along with the volume of diluent to determine the concentration of each component. For the purposes of these analyses, APIs were categorized into eight different types including monoclonal antibodies, antibody conjugates, enzymes, cytokines and growth factors, polypeptide hormones, pulmonary surfactants (phospholipoproteins that reduce alveolar surface tension), recombinant fusion proteins, and toxins. Antibody conjugate molecule types included antibody-drug conjugates, antibody linker-chelator conjugates and antibody-toxin conjugates.

### Drug Product Attributes and their Categorization

With a few exceptions, biotechnology-derived drug products are mainly delivered through the parenteral route and formulated as stable liquids or powder for reconstitution. In some cases, further dilution is required for patient-specific dosing or administration through an alternate route. Physical storage conditions such as the defined stable storage period or temperature can be different based on the presentation status of a drug formulation, whether it has been reconstituted or diluted. If a product requires reconstitution or dilution prior to the indicated route of administration, we recorded the appropriate diluent and volume that yielded the maximum allowable concentration of the active ingredient, per the label instructions. Appropriate route of administration and other physicochemical stability attributes including the pH range, requirements to protect from light, and indication not to freeze or shake the product were also recorded for the analyses.

### Consistency of Chemical Terminologies

In order to perform consistent correlations with the data, various analogous terms used in drug labels for the same component of the drug product were evaluated and consolidated into a common term in the database prior to performing queries. The FDA’s Substance Registration System (SRS) provides unique identifiers for substances used in drugs, biologics, foods and devices based on molecular structure and descriptive information and was used as a reference (9). After consolidation of different excipient terms using SRS, a variation of hydrated forms and salt forms of ionic compounds were further consolidated into general chemical terms used in PubChem (10). Each excipient registered in the system has a Unique Ingredient Identifier (UNII) and the SRS list of synonyms was used for mapping the same excipient.

### Categorization of Excipients and Drug Products for Analyses

Based on the protein API and context of use, a given excipient could have distinct roles in different formulations or have multiple roles in a single formulation. Therefore, when the package insert lacked clear information on the intended use, excipient monographs from the Handbook of Pharmaceutical Excipients were used to categorize each excipient based on its primary proposed physicochemical function in the appropriate dosage form (11). If an excipient monograph was unavailable for a listed excipient in the Handbook, the Encyclopedia of Pharmaceutical Technology was utilized as a secondary resource (12). We acknowledge that excipients have multiple roles, even within the same formulation, and our analyses should not be construed as an attempt to restrict the interpretation or application of these excipients. For the purposes of this analysis, excipients were categorized into 14 different functional categories (Table III). In some instances, excipients with an undetermined role based on available resources were categorized as ‘other’ and are designated as such in the findings. Prevalence of excipients among drug formulations was determined by these functional categories and the context in which they were presented for each drug product surveyed. Graphs were generated using the Prism 6 for Windows v6.07(GraphPad Software, LaJolla, CA).

## Results

### Creating a Dataset of Excipients Used in Biotechnology-Derived Drug Products

There are 211 biotechnology-derived APIs currently approved by CDER and marketed in the U.S. in 665 different formulations. Of 665 original formulations, 397 formulations were considered unique based on having unique excipients. The active ingredients in these formulations were categorized by molecular class, including antibody conjugates, cytokine and growth factors, enzymes, monoclonal antibodies, polypeptide hormones, pulmonary surfactants, recombinant fusion proteins, and toxins. The number of formulations under each category and dosage form are listed in Table I. Most biotechnology-derived drug products are administered through parenteral routes and manufactured as either liquids or powder for reconstitution. However, there were 13 formulations available as either a solid dosage form for oral administration or semisolid dosage forms for topical administration. Among different molecule types, monoclonal antibodies with 118 formulations were the most populous group, followed by polypeptide hormones with 109 formulations and cytokines and growth factors with 83 formulations.

**Table 1:** Number of Unique Formulations by Molecule Types of Active Pharmaceutical Ingredients and Dosage Forms

Molecule type	Powder for recon.	Liquid	Solid^1^	Semisolid^2^	Total
Antibody conjugate^3^	6	2			8
Cytokine and growth factor	20	62		1	83
Enzyme	17	25	5	1	48
Monoclonal antibody^4^	26	92			118
Polypeptide hormone	48	55	6		109
Pulmonary surfactant		3			3
Recombinant fusion protein	8	10			18
Toxin	9	1			10
Total	134	250	11	2	397

^1^Solid dosage forms include tablet, capsule and powder for inhalation

^2^Semisolid dosage forms include gel and ointment

^3^Antibody conjugate molecule types include antibody-drug conjugates, antibody linker-chelator conjugate, and antibody-toxin conjugate

^4^Includes antibody fragments and bispecifics

**Fig. 1:**
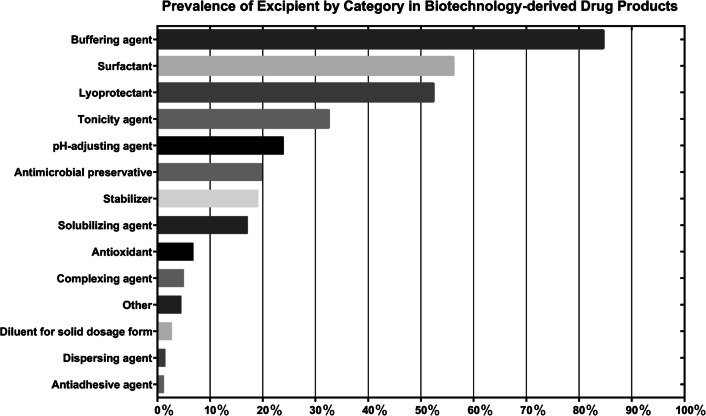
Prevalance of excipient by category in biotechnology-derived drug products

**Fig. 2:**
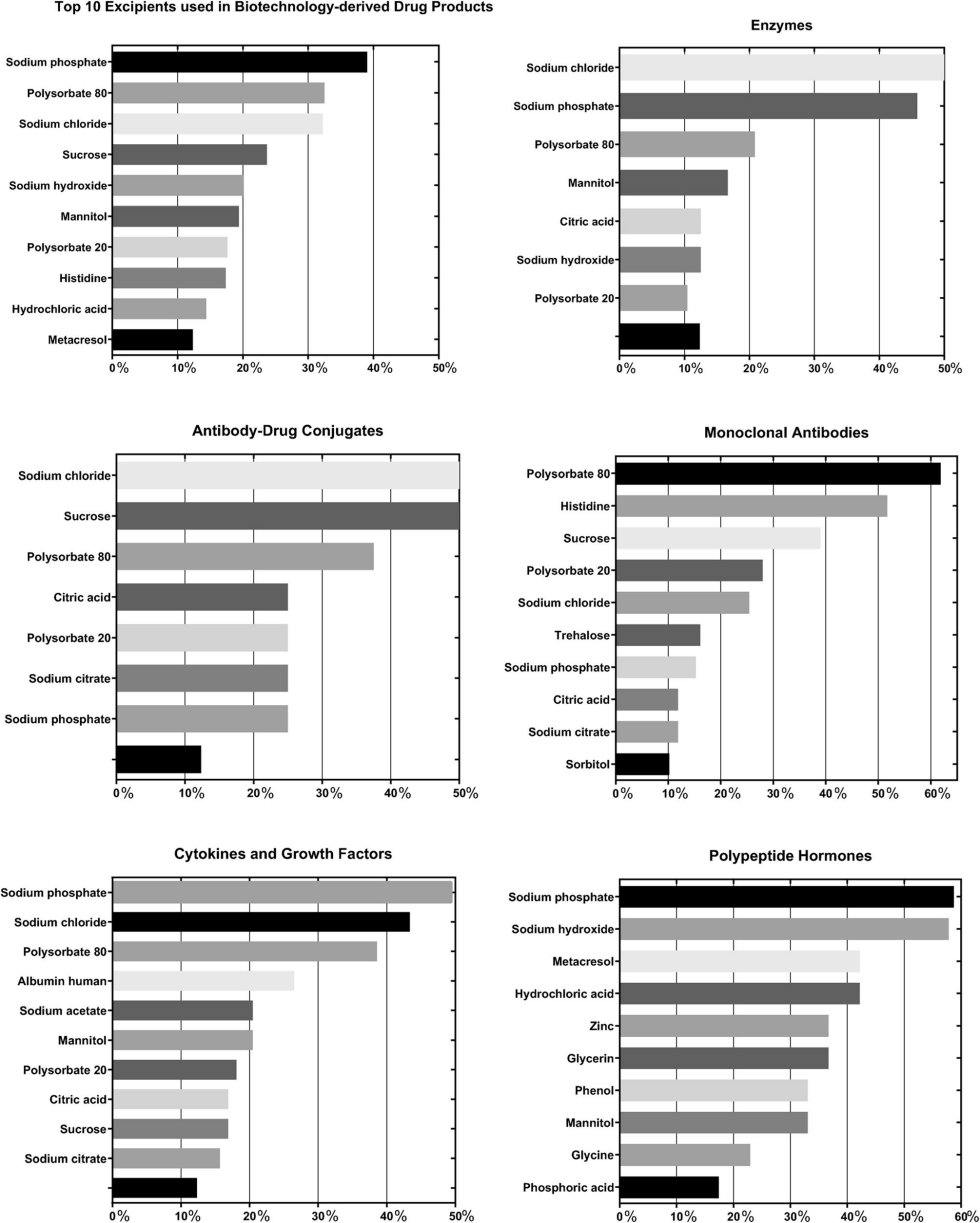

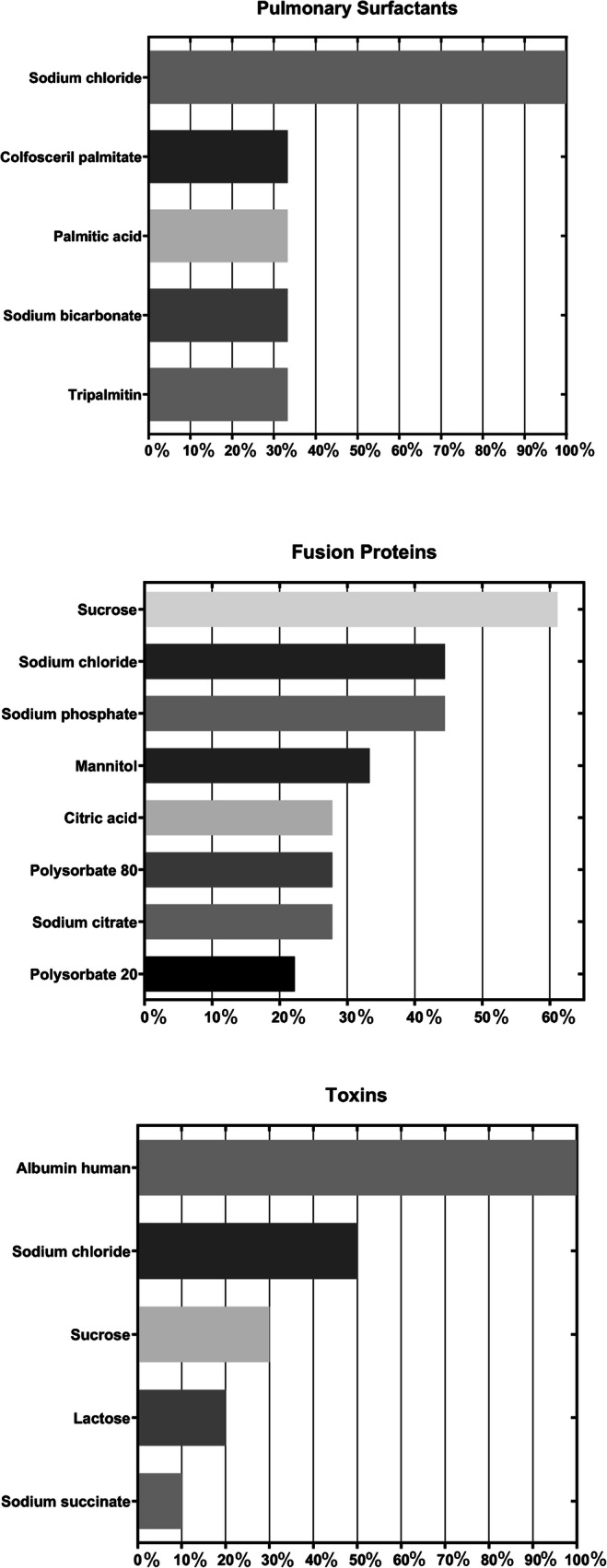
Prevalence of excipients among unique formulations for different molecule type of active pharmaceutical ingredients

Excipient categories and individual excipients were first counted for each molecule type of active ingredients, and prevalence was calculated as a percentage based on the total number of formulations for each molecule type, shown in Table II. Commonly found excipient categories include buffering agents (84.63%), surfactants (56.17%), lyoprotectant (52.39%), tonicity agents (32.49%), and pH-adjusting agents (23.93%), as shown in order of most to least prevalent in Fig. 1 (Table III). For individual excipients, a total of 96 distinct excipients were identified, and prevalence was calculated for all formulations (Table IV) and for each molecule type (Table V). The data are also graphically represented in Fig. 2 for each of the different molecule types. Common excipients across all molecule types in original formulations were sodium phosphate (39.04%), polysorbate 80 (32.49%), sodium chloride (32.24%), sucrose (23.68%), and sodium hydroxide (20.15%), which function as buffering agents, surfactants, tonicity agents, lyoprotectants and pH-adjusting agent, respectively.

**Table 2:** Prevalence of Excipient Categories by Molecule Type of Active Pharmaceutical Ingredients

Excipient category	Antibody conjugate (*n* = 8)	Cytokine and growth factor (*n* = 83)	Enzyme (*n* = 48)	Monoclonal antibody (*n* = 118)	Polypeptide hormone (*n* = 109)	Pulmonary surfactant (*n* = 3)	Recombinant fusion protein (*n* = 18)	Toxin (*n* = 10)	Total (*n* = 397)
Buffering agent	87.50%	96.39%	77.08%	96.61%	72.48%	0.00%	100.00%	10.00%	84.63%
Surfactant	62.50%	67.47%	33.33%	93.22%	22.94%	33.33%	55.56%	0.00%	56.17%
Lyoprotectant	62.50%	44.58%	29.17%	65.25%	50.46%	0.00%	83.33%	50.00%	52.39%
Tonicity agent	50.00%	43.37%	52.08%	25.42%	16.51%	100.00%	44.44%	50.00%	32.49%
pH-adjusting agent	12.50%	4.82%	25.00%	8.47%	60.55%	33.33%	5.56%	0.00%	23.93%
Antimicrobial preservative	0.00%	19.28%	10.42%	0.00%	53.21%	0.00%	0.00%	0.00%	19.90%
Stabilizer	0.00%	26.51%	8.33%	0.00%	36.70%	0.00%	0.00%	100.00%	19.14%
Solubilizing agent	0.00%	14.46%	4.17%	10.17%	36.70%	0.00%	11.11%	0.00%	17.13%
Antioxidant	0.00%	12.05%	2.08%	7.63%	6.42%	0.00%	0.00%	0.00%	6.80%
Complexing agent	0.00%	3.61%	6.25%	11.02%	0.00%	0.00%	5.56%	0.00%	5.04%
Other	0.00%	15.66%	10.42%	0.00%	0.00%	0.00%	0.00%	0.00%	4.53%
Diluent for solid dosage form	0.00%	0.00%	10.42%	0.00%	5.50%	0.00%	0.00%	0.00%	2.77%
Dispersing agent	0.00%	2.41%	0.00%	3.39%	0.00%	0.00%	0.00%	0.00%	1.51%
Antiadhesive agent	0.00%	6.02%	0.00%	0.00%	0.00%	0.00%	0.00%	0.00%	1.26%

*Subcategories for diluent for solid dosage forms include drug powder inhaler carrier, coating agent, plasticizing agent, glidant, disintegrant, lubricant, binder and water-repelling agent

**Table 3:** Categories of Excipients Found in Biotechnology-derived Drug Products and Examples of Excipients

Excipient category^*^	Examples
Antiadhesive agent	isoleucine
	leucine
Antimicrobial preservative	benzyl alcohol
	calcium chloride
	metacresol
	methylparaben
	phenol
	propylparaben
	thimerosal
Antioxidant	methionine
	niacinamide
Buffering agent	acetate
	acetic acid
	alanine
	arginine
	aspartic acid
	citric acid
	glutamic acid
	histidine
	lysine
	potassium phosphate
	sodium acetate
	sodium citrate
	sodium phosphate
	sodium succinate
	succinic acid
	tromethamine
Complexing agent	calcium acetate
	edetate disodium
	edetic acid
	pentetic acid
	stannous chloride
Dispersing agent	carboxymethylcellulose sodium
	hypromellose
	proline
Lyoprotectant	dextran 40
	glycine
	lactose
	mannitol
	sucrose
	trehalose
pH-adjusting agent	hydrochloric acid
	lactic acid
	phosphoric acid
	sodium bicarbonate
	sodium carbonate
	sodium hydroxide
Solubilizing agent	glycerin
	polyethylene glycol 3350
	polyethylene glycol 6000
	sorbitol
Stabilizer	albumin human
	protamine sulfate
	zinc^**^
Surfactant	colfosceril palmitate
	palmitic acid
	poloxamer 188
	polysorbate 20
	polysorbate 80
	sodium lauryl sulfate
	tripalmitin
Tablet and capsule diluent	cellacefate
	cellulose
	cetyl alcohol
	croscarmellose sodium
	crospovidone
	diethyl phthalate
	dimethicone
	fumaryl diketopiperazine
	hydrogenated castor oil
	hypromellose phthalate
	magnesium stearate
	mantan wax
	methacrylic acid copolymer
	microcrystalline cellulose
	n-vinylpyrrolidinone
	polyethylene glycol
	silicon dioxide
	simethicone
	sodium starch glycolate
	stearic acid
	talc
	triethyl citrate
Tonicity agent	dextrose
	guanidine
	magnesium chloride
	maltose
	potassium chloride
	sodium chloride
Other	cinnamic acid
	petrolatum
	phenylalanine
	sodium sulfate
	threonine
	tranexamic acid
	ursodiol

^*^Definitions per Handbook of Pharmaceutical Excipients, 2020 (Reference# 11)

^**^Zinc was present either as a result of co-crystallization and/or supplemented by addition of zinc compounds such as zinc chloride, acetate or oxide

**Table 4:** Common Excipients Among Unique Formulations for All Biotechnology-derived Drug Products

Excipients	Percentage of unique formulations (*n* = 397)
Sodium phosphate	39.04%
Polysorbate 80	32.49%
Sodium chloride	32.24%
Sucrose	23.68%
Sodium hydroxide	20.15%
Mannitol	19.40%
Polysorbate 20	17.63%
Histidine	17.38%
Hydrochloric acid	14.36%
Metacresol	12.34%

**Table 5:** Common Excipients Among Unique Formulations for Different Molecule Type of Active Pharmaceutical Ingredients

**Excipients**	**Monoclonal Antibody (*n* = 118)**	**Excipients**	**Polypeptide Hormone (*n* = 109)**
Polysorbate 80	61.86%	sodium phosphate	58.72%
Histidine	51.69%	sodium hydroxide	57.80%
Sucrose	38.98%	hydrochloric acid	42.20%
Polysorbate 20	27.97%	metacresol	42.20%
Sodium chloride	25.42%	glycerin	36.70%
Trehalose	16.10%	zinc	36.70%
Sodium phosphate	15.25%	mannitol	33.03%
Citric acid	11.86%	phenol	33.03%
Sodium citrate	11.86%	glycine	22.94%
Sorbitol	10.17%	phosphoric acid	17.43%
**Excipients**	**Cytokine and Growth Factor (*n* = 83)**	**Excipients**	**Enzyme (*n* = 48)**
Sodium phosphate	49.40%	sodium chloride	50.00%
Sodium chloride	43.37%	sodium phosphate	45.83%
Polysorbate 80	38.55%	polysorbate 80	20.83%
Albumin human	26.51%	mannitol	16.67%
Mannitol	20.48%	citric acid	12.50%
Sodium acetate	20.48%	sodium hydroxide	12.50%
Polysorbate 20	18.07%	polysorbate 20	10.42%
Sucrose	16.87%		
Citric acid	16.87%		
Sodium citrate	15.66%		
**Excipients**	**Recombinant Fusion Protein (*n* = 18)**	**Excipients**	**Antibody Conjugate (*n*** **= 8)**
Sucrose	61.11%	sodium chloride	50.00%
Sodium chloride	44.44%	sucrose	50.00%
Sodium phosphate	44.44%	polysorbate 80	37.50%
Mannitol	33.33%	citric acid	25.00%
Citric acid	27.78%	polysorbate 20	25.00%
Polysorbate 80	27.78%	sodium citrate	25.00%
Sodium citrate	27.78%	sodium phosphate	25.00%
Polysorbate 20	22.22%		
**Excipients**	**Toxin (*n* = 10)**	**Excipients**	**Pulmonary Surfactant (*n* = 3)**
Albumin human	100.00%	sodium chloride	100.00%
Sodium chloride	50.00%	colfosceril palmitate	33.33%
Sucrose	30.00%	palmitic acid	33.33%
Lactose	20.00%	sodium bicarbonate	33.33%
Sodium succinate	10.00%	tripalmitin	33.33%

Among commonly found excipients, the maximum used concentration of 1.6% was noted for sodium phosphate in a formulation that was administered via intramuscular route. Polysorbate 80 and polysorbate 20 were mostly found at a lower than 0.1% concentration, but polysorbate 80 was found at a maximum concentration of 0.65% in an antibody conjugate formulation administered intravenously. Polysorbate 20 was noted at a maximum concentration of 0.2% in a polypeptide hormone administered subcutaneously. Sucrose was noted at a broad range of concentrations from 0.001 to 20%, with the highest and lowest concentrations observed with recombinant fusion protein formulations. Other sugar excipients such as trehalose were noted at a relatively higher concentrations with a maximum concentration of 10.4%, mannitol up to 10%, maltose up to 5%, and lactose up to 2.3%. For sodium chloride, the maximum concentration observed was 1.169% in a monoclonal antibody formulation administered subcutaneously. For sodium hydroxide, when added for pH adjustment and the quantity was stated in the prescribing information, a maximum concentration of 0.0523% was noted in a formulation administered intravenously.

Excipients unique to solid dosage forms were found in enzyme replacement products formulated as tablets or capsules, and insulin developed for inhalation. These excipients are only present in 11 formulations out of 397 unique formulations, and specific subcategories of these excipients were grouped into a single category of “diluent for solid dosage form”. Some of the subcategories of excipients for solid dosage forms included drug powder inhaler carrier, coating agent, plasticizing agent, glidant, disintegrant, lubricant, binder, and water-repelling agent.

### Correlation with Route of Administration and Recommended pH Range

Based on the analyses, most drugs were formulated with the intent of being administered through subcutaneous, intravenous or intramuscular routes. In some instances, a single product was administered through multiple routes of administration or further diluted to allow an alternate route of administration. Table VI shows the number of formulations for each route of administration as indicated in the PI. The PI also provides the pH range for parenteral solutions. The maximum and minimum pH of formulations grouped according to the intended route of administration is listed in Table VII.

**Table 6:** Number of Formulations by Route of Administration

Route of administration	Number of formulations
Infiltration	3
Interstitial	3
Intracatheter	1
Intradermal	3
Intradetrusor	3
Intraglandular	4
Intralesional	3
Intramuscular	33
Intraocular	3
Intratracheal	3
Intravenous	154
Intraventricular	1
Intravitreal	5
Ophthalmic	1
Oral	6
Peribulbar	2
Respiratory (inhalation)	7
Retrobulbar	3
Soft tissue	3
Subcutaneous	249
Topical	2
Total	492*

*The total number of formulations is larger than the number of unique formulations (397) because a single formulation can have more than one route of administration

**Table 7:** pH Ranges of Formulations for Different Route of Administration

Route of administration	pH range
Infiltration	6.4–7.2
Interstitial	6.4–7.2
Intracatheter	7.3
Intraglandular	5.6
Intramuscular	4.8–8
Intraocular	6.4–7.2
Intratracheal	5–6.5
Intravenous	5–8
Intraventricular	6.2–6.8
Intravitreal	3.1–7.2
Ophthalmic	7–7.4
Oral	4–4.7
Peribulbar	6.4–7.2
Respiratory (inhalation)	6.3
Retrobulbar	6.4–7.2
Soft tissue	6.4–7.2
Subcutaneous	4–9
Topical	6–8

### Commonalities and Trends in the Excipients Used

Based on our survey, the three functional categories of most commonly used excipients in all biotechnology-derived products were buffering agents, surfactants, and lyoprotectants. These excipients are necessary to stabilize pH, reduce surface and interfacial tension and prevent damage during the lyophilization process, especially in the presence of a protein API that is susceptible to misfolding and conformational changes. The most common buffering agents used included sodium phosphate, histidine, citric acid, sodium citrate, and sodium acetate. While sodium phosphate appears as a common ingredient, it has several noteworthy limitations arising from phosphate-related issues, propensity for aluminum-containing particulates, and pH shifts resulting in destabilizing crystallization during freezing (13,14). The most commonly used surfactants included polysorbate 80, polysorbate 20, and poloxamer 188. These agents are used not only to stabilize against interfacial tension but also to reduce aggregation or protein-protein interactions. The most commonly used lyoprotectants included sucrose, mannitol, and glycine. Among the 255 formulations that contained a surfactant, 129 contained polysorbate 80 and 70 contained polysorbate 20. Polaxamer 188 was reported in 20 formulations. Excluding pulmonary surfactants and toxins which accounted for 13 formulations in our survey , buffering agents were present in high prevalence in over 70% of all drug molecule types. Other than commonly found agents mentioned, buffering agents had variety of agents included in its category including acetate containing buffers (13.6% combined) and succinate buffers (3.02% combined). Sodium phosphate, which is the most commonly found excipients in all drug formulations, is listed as the most used buffering agent with each molecule type, excluding monoclonal antibodies. Monoclonal antibodies were commonly formulated with amino acid excipients for stabilization purposes, and histidine was noted as the most common buffering agent for monoclonal antibodies (15). Histidine was present in 69 formulations across all drug types, and of those formulations, 61 formulations were monoclonal antibodies. Sodium phosphate was the second most prevalent buffering agent in monoclonal antibodies with 15% of monoclonal antibodies formulations containing this excipient. Lyoprotectants, such as sucrose and trehalose were also commonly found in monoclonal antibody formulations used to optimize protein stability and minimize extent of protein aggregation, especially for those drugs that are lyophilized . In drugs with sucrose (94 formulations), 46 were monoclonal antibodies and eight were antibody conjugates. Across all drug formulations with trehalose (23 formulations), 19 formulations were monoclonal antibodies and one formulation was an antibody conjugate.

Buffering agents were also present in high prevalence (96.39%) in cytokine and growth factor drug formulations. A stabilizer and solubilizing agent, such as albumin, is also one of the common excipients found in cytokine and growth factor formulations with a prevalence of 26.51%. Albumin was found in 35 formulations across all molecule types, and of those, 22 formulations were cytokine and growth factors, 10 toxins and three enzymes. The addition of albumin likely also reflects the product-specific need for greater molecular stability, reduced exposure of the protein API surface, and reduced oxidation susceptibility while maintaining desired half-life. Although toxins accounted for only 10 drug formulations, lyoprotectants and stabilizers such as sucrose and albumin were found in all toxin formulations.

### Some Excipients Unique to Protein Product Presentations

Antimicrobial preservatives were found in protein formulations that were intended to deliver multiple doses and included polypeptide hormones, enzyme, and cytokines and growth factors drug formulations. More than half of polypeptide hormone formulations, which included insulin products, contained antimicrobial preservatives, such as metacresol (42.2%) and phenol (33.03%). Phenol was noted in some polypeptide hormone formulations, while metacresol was noted in cytokine and growth factor or polypeptide hormone formulations. Among the formulations with metacresol, the majority (46 of 49) were polypeptide hormones. Zinc and protamine sulfate were added to insulin as stabilizers that form complexes and control the duration of action (12). Zinc was present either as a result of co-crystallization, in examples with insulin, and/or supplemented with either zinc oxide, zinc acetate or zinc chloride in 41 formulation. Protamine sulfate was reported in 17 insulin formulations. There were also other unique excipients only found in enzyme and polypeptide hormone formulations such as glycerin, a solubilizing agent. Phosphoric acid, used to adjust pH of formulations, was found in 19 polypeptide hormone and four enzyme formulations.

## Discussion

In this report, we provide a comprehensive survey of excipients used in a wide range of biotechnology derived drug products. We present a scientific assessment of the common features and trends in excipient use across multiple sub-types of therapeutic proteins. Comparison of all excipients within the different classes of excipients also points to certain unique excipients used in protein formulations such as amino acids, lyoprotectants, and solubilizers. We envision that our evidence-driven analyses can be utilized as a reference to research excipients that have been applied to large protein molecular APIs and facilitate the pre-formulation efforts by new and experienced drug developers. The survey on prevalence of excipients across drug types is also aimed at providing the data on the extent to which a specific excipient or type of excipient is used in multiple protein products. This information should assist drug developers with performing a more pragmatic risk assessment of product quality problems or adverse reactions linked to a specific excipient or type of excipient. We acknowledge that the inclusion and categorization of excipients used for the purposes of our survey is subject to different interpretations and applications under different formulating conditions. Many excipients have multiple roles, even within the same formulation, and dependent on the context of use. The information provided should also offer a reference point for protein-specific formulation studies when developing new drug products or new formulations of existing drug products. Additionally, our report provides insights based on a comprehensive analysis of drug formulation attributes such as composition, ingredient strength, route of administration, and pH, which can aid in performing risk assessment of incidences and pathways of protein instability and aggregation that might be linked to these specific product attributes. We also note that, despite the numerous excipient categories being represented, the portfolio of excipients for biologics is somewhat limited considering the rapid pace of protein engineering resulting in novel molecules, novel product presentations, non-routine routes of administration for proteins, and a wide range of in-use conditions and delivery systems for these drugs. Therapeutic protein formulations with novel stabilizers that do not require refrigeration or conservative storage conditions of temperature/light/humidity could allow flexible transport and rapid deployment of such drugs across diverse environmental conditions. Similarly, excipients that allow consistent high quality and safe delivery of therapeutic proteins across the blood-brain barrier, by aerosolized delivery, or for topical application could also meet unmet needs. We envision that our report can serve as a resource for future studies that aim to develop stable and high-quality drug products, or novel excipients that offer superior performance compared to existing excipients found in biotechnology-derived drug products and facilitate the rapid development of new drug molecules.

## ABBREVIATIONS

NDC: National drug code
SRS: Substance registration system
UNII: Unique ingredient identifier
