# Correction to: A Comprehensive Scientific Survey of Excipients Used in Currently Marketed, Therapeutic Biological Drug Products

**DOI:** 10.1007/s11095-022-03253-7

**Published:** 2022-04-15

**Authors:** V. Ashutosh Rao, Jennifer J. Kim, Dipti S. Patel, Kimberly Rains, Corey R. Estoll

**Affiliations:** 1 Laboratory of Applied Biochemistry, Division of Biotechnology Review and Research III, Office of Biotechnology Products, Center for Drug Evaluation and Research, Food and Drug Administration, 10903 New Hampshire Ave / Bldg. 52/72 Rm 2212, Silver Spring, Maryland 20993 USA; 2 Present Address: Office of Biotechnology Products, Center for Drug Evaluation and Research, Food and Drug Administration, Silver Spring, MD USA; 3 University of Maryland School of Pharmacy, Baltimore, Maryland USA; 4 Office of Biotechnology Products, Center for Drug Evaluation and Research, Food and Drug Administration, Silver Spring, Maryland USA

**Correction to: Pharm Res**

**https://doi.org/10.1007/s11095-020-02919-4**

The article “A Comprehensive Scientific Survey of Excipients Used in Currently Marketed, Therapeutic Biological Drug Products,” written by V. Ashutosh Rao, Jennifer J. Kim, Dipti S. Patel, Kimberly Rains, and Corey R. Estoll, was originally published Online First without Open Access. After publication in volume 37, issue 10, the authors decided to opt for Open Choice and to make the article an Open Access publication. Therefore, the copyright of the article has been changed to © The Author(s) 2022 and the article is forthwith distributed under the terms of the Creative Commons Attribution 4.0 International License, which permits use, sharing, adaptation, distribution and reproduction in any medium or format, as long as you give appropriate credit to the original author(s) and the source, provide a link to the Creative Commons licence, and indicate if changes were made. The images or other third party material in this article are included in the article’s Creative Commons licence, unless indicated otherwise in a credit line to the material. If material is not included in the article’s Creative Commons licence and your intended use is not permitted by statutory regulation or exceeds the permitted use, you will need to obtain permission directly from the copyright holder. To view a copy of this licence, visit http://creativecommons.org/licenses/by/4.0

